# A lightweight defect detection algorithm for escalator steps

**DOI:** 10.1038/s41598-024-74320-9

**Published:** 2024-10-11

**Authors:** Hui Yu, Jiayan Chen, Ping Yu, Da Feng

**Affiliations:** 1 College of Energy Environment and Safety Engineering & College of Carbon Metrology, China Jiliang University, Hangzhou, 310018 China; 2 College of Quality and Standardization, China Jiliang University, Hangzhou, 310018 China; 3 Huzhou Special Equipment Inspection Center, Huzhou, 313099 China

**Keywords:** Computer science, Information technology

## Abstract

In this paper, we propose an efficient target detection algorithm, ASF-Sim-YOLO, to address issues encountered in escalator step defect detection, such as an excessive number of parameters in the detection network model, poor adaptability, and difficulties in real-time processing of video streams. Firstly, to address the characteristics of escalator step defects, we designed the ASF-Sim-P2 structure to improve the detection accuracy of small targets, such as step defects. Additionally, we incorporated the SimAM (Similarity-based Attention Mechanism) by combining SimAM with SPPF (Spatial Pyramid Pooling-Fast) to enhance the model’s ability to capture key information by assigning importance weights to each pixel. Furthermore, to address the challenge posed by the small size of step defects, we replaced the traditional CIoU (Complete-Intersection-over-Union) loss function with NWD (Normalized Wasserstein Distance), which alleviated the problem of defect missing. Finally, to meet the deployment requirements of mobile devices, we performed channel pruning on the model. The experimental results showed that the improved ASF-Sim-YOLO model achieved an average accuracy (mAP50) of 96.8% on the test data set, which was a 22.1% improvement in accuracy compared to the baseline model. Meanwhile, the computational complexity (in GFLOPS) of the model was reduced to a quarter of that of the baseline model, while the frame rate (FPS) was improved to 575.1. Compared with YOLOv3-tiny, YOLOv5s, YOLOv8s, Faster-RCNN, TOOD, RTMDET and other deep learning-based target recognition algorithms, ASF-Sim-YOLO has better detection accuracy and real-time processing capability. These results demonstrate that ASF-Sim-YOLO effectively balances lightweight design and performance improvement, making it highly suitable for real-time detection of step defects, which can meet the demands of escalator inspection operations.

## Introduction

With the continuous advancement of urbanisation in China, the domestic elevator industry has experienced significant growth. By the end of 2022, the total number of specialised equipment in China had reached 195.25 million units, including 9,644,600 elevators. Escalators, crucial for transporting large numbers of people in shopping malls, subway stations, and other public spaces, have seen a rapid increase in ownership in recent years^1,2^. However, along with this rise, there has also been an increase in accidents^3^.

The operational environment of escalators, characterised by high foot traffic, accelerates the wear and tear process, leading to a higher failure rate^4^. Escalator malfunctions can lead to serious accidents, such as pinching and falls, putting passengers’ lives at risk. Therefore, precise and timely detection and identification of escalator defects are crucial.

The absence of teeth on escalator steps can create gaps in movement, increasing the risk of accidents such as pinching and heightening the likelihood of passenger injuries^5^. Currently, the detection of escalator step defects relies primarily on visual inspection by maintenance personnel, which may not promptly and accurately identify safety hazards. Manual inspections are prone to overlooking minor defects or issues that are challenging to detect, incurring substantial time and labour costs. Hence, there is a pressing need for a more efficient and accurate automated inspection method to address the limitations of traditional approaches.

### Related work

In recent years, there has been a growing trend in applying image processing techniques to surface defect detection. Bhatt used a gradient thresholding method to identify defects in cabinets^6^. This method involves comparing the grey values of defective areas with the background to detect anomalies. Similarly, in a separate study by Ali, a combination of a threshold selection filter and the K-means algorithm (a clustering method)^7^ was proposed. This was followed by filtered image segmentation to precisely delineate the boundaries of blind holes and cracks in industrial materials. With the rapid advancement of computer vision and deep learning techniques, target detection methods have become widely adopted across various domains. These techniques enable rapid and accurate identification and recognition of targets in images or videos, providing significant advantages. Consequently, integrating these sophisticated techniques into the realm of escalator defect detection can substantially enhance detection accuracy and efficiency.

Presently, mainstream target detection algorithms fall into two categories: one-stage^8^ and two-stage^9^ methods. One-stage algorithms typically generate candidate frames and simultaneously perform classification and bounding box regression. While they exhibit fast processing speeds, they often suffer from lower accuracy. Examples include the YOLO^10^ family of algorithms and the SSD^11^ algorithm.

On the other hand, two-stage target detection algorithms follow a sequential process. They first generate candidate frames for the region and extract features from each candidate frame. Subsequently, they generate positional frames and predict corresponding categories. Notable examples include Fast R-CNN^12^, Faster R-CNN^13^, and FPN^14^ algorithms. While these methods offer higher accuracy, they tend to operate at slower speeds.

Liu presented a study on the detection and identification of escalator step defects using machine vision^15^, while Ma introduced a study and application focusing on escalator defect image recognition^16^ based on YOLOv3. Target detection technology has a variety of applications. Compared to humans, computers have a significant speed advantage in handling target detection tasks, thereby greatly enhancing work efficiency. This involves using defect detection algorithms to identify equipment defects and overlaying defect information onto real equipment using augmented reality. Zhang proposed an algorithm for industrial defect detection^17^ based on YOLOv8, aiming to improve feature detection accuracy for small targets. Additionally, Wang introduced a new detection model, YOLO-SAGC^18^, which enhances feature recognition by integrating self-attention^19^ and graphical convolution in the head module. Another aspect involves a comprehensive optimisation of the entire network architecture using a lightweight module in combination with depth-separable convolution. Furthermore, Wang proposed a channel pruning-based method for small target detection^20^ in YOLOv5s, effectively simplifying the model while ensuring detection accuracy.

As inspection processes increasingly transition towards automation, edge devices such as UAV^21^, robots, and PDAs have emerged as pivotal tools. However, these devices are constrained by limited computing power and battery capacity. Hence, single-stage detectors, characterised by low computational requirements and high detection efficiency, are better suited for edge device scenarios. In the case of single-stage detectors, the YOLO series is notable for eliminating the region proposal network and improving detection efficiency. This approach entails performing object-class classification and bounding box regression directly on a complete image. Despite their faster processing speeds compared to two-stage detectors, most single-stage detectors still struggle to achieve satisfactory results on edge devices. This is attributed to several factors. Firstly, the current computational power of devices remains relatively low, hindering single-stage detectors from achieving faster detection speeds. Furthermore, single-stage detectors’ detection accuracy is often insufficient for practical applications.

The lightweight version of the single-stage detector mostly keeps the same core architecture as the original version. However, it uses smaller convolutional kernels^22^, has fewer convolutional layers, and doesn’t have as many feature extraction branches. This approach substantially reduces the number of parameters and enhances computational speed^23^. However, it often results in inadequate feature extraction and insufficient feature fusion, leading to a notable decline in detection accuracy.

There are also a lot of different use cases and variations in the computing power of the devices doing the detection task, which makes it hard to use existing algorithms on mobile devices to meet the needs of real-time detection. These difficulties arise because of the diverse computational resources available across different devices and the specific demands of real-time detection in dynamic environments^24^.

Enabling deep learning models to accurately locate and detect regions of interest, reducing noise interference, and improving model robustness is another key factor affecting detection accuracy. Therefore, achieving a balance between accuracy and speed on a one-level detector becomes a key research point for algorithm application on edge devices.

However, insufficient samples are currently a common problem in the field of defect detection, especially in escalator step defect detection. The high level of difficulty in image data collection and the lack of focus on escalator step defects has led to a scarcity of available public datasets.

### Our approach

To address the above problems, we made a series of improvements to YOLOv8n and designed a new model named ASF-Sim-YOLO. In ASF-Sim-YOLO, we proposed a new detection framework ASF-P2, and at the same time, we improved the SPPF module by incorporating the SimAM attention mechanism. We used the NWD loss function to replace the default CIoU loss function of YOLOv8 for the distribution and scale characteristics of escalator step defects, and we adopted the LAMP method to prune the model. These enhancements resulted in higher accuracy and a more lightweight model. In summary, the main contributions of this study are as follows:

1. A new detection model ASF-Sim-YOLO was designed, and the P2 small target detection layer was introduced for the characteristics of escalator step defects, which effectively improved the detection accuracy.
2. The inclusion of the SimAM attention mechanism in Spatial Pyramid Pooling-Fast (SPPF) enhanced the extraction of defective features without increasing the number of parameters, improved the model’s ability to detect defects, and reduced the background noise that interferes with detection.
3. We replaced the default CIoU of YOLOv8 with the NWD loss function for defects to solve the problems of inaccurate recognition of small and dense defects at the far end of the image and inaccurate recognition of defects at the edges of the image, for example.
4. The problem of high parametric counts and high inference latency of the model was improved by pruning the channels according to the weight scores, which also made the model lighter.

## Methodologies

### Methods overview

In this paper, we propose an escalator step defect detection model based on YOLOv8, tailored to the specific characteristics of escalator step defects. The YOLOv8 framework includes five scales: YOLOv8n, YOLOv8s, YOLOv8m, YOLOv8l, and YOLOv8x. Given the stringent real-time and low-latency requirements for deployment on mobile devices, we selected the lightest model, YOLOv8n, for further enhancement and named it ASF-Sim-YOLO.

**Fig. 1:**
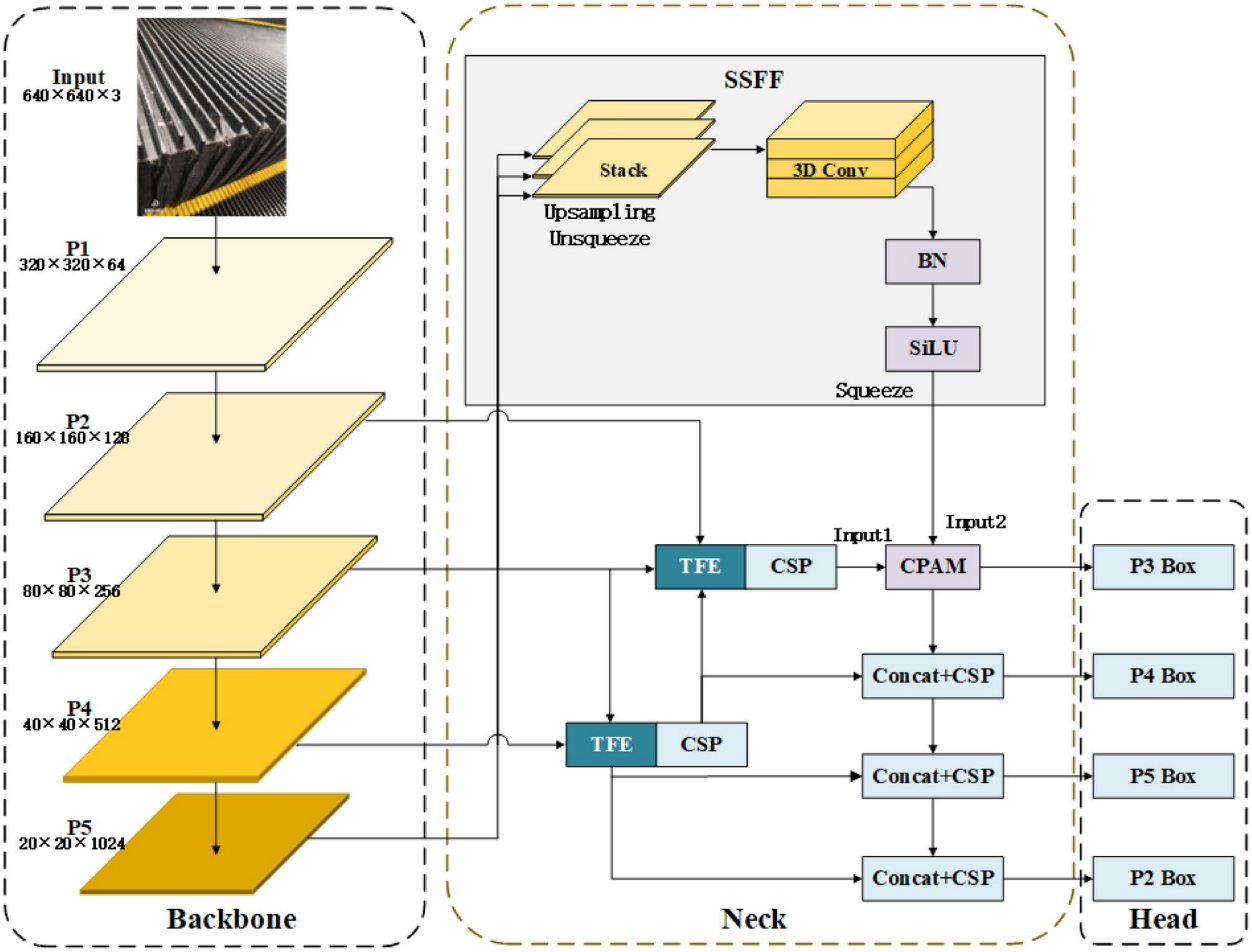
ASF-Sim-P2 framework.

**Fig. 2:**
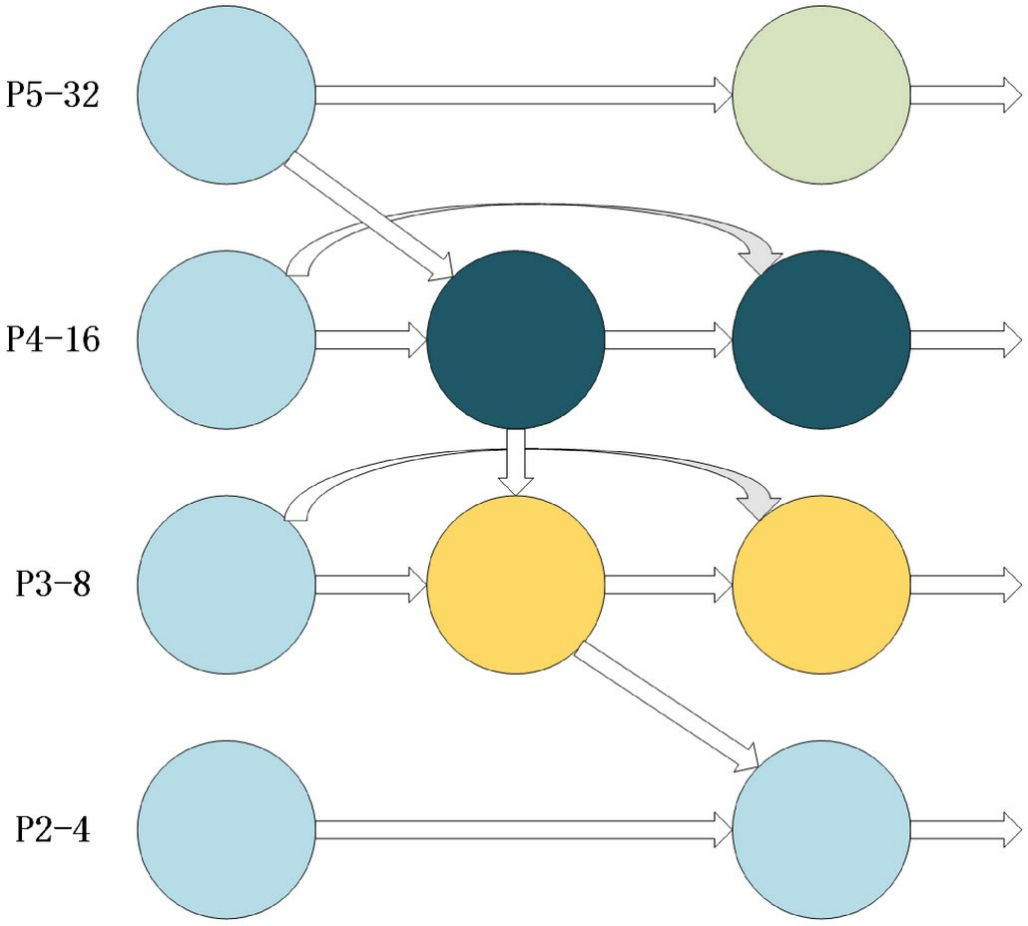
Added P2 small target detection layer.

The structure of the designed model network is shown in Figs. 1 and 2. Most of the escalator step defects are in the form of missing teeth, which are characterised by a small scale, a high degree of randomness in the location of occurrence, and a lack of clear distinction between the defects and the normal part.

Kang introduced the ASF-YOLO^25^ framework, for instance, for segmentation of images, incorporating spatial and multi-scale features^26^. The framework comprises two primary components:

1. the SSFF module: Consolidates global or high-level semantic information from multi-scale images.
2. the TFE module: Captures detailed information about small objects.

By amalgamating local and global feature information, more precise segmentation maps can be generated. Initially, the framework combines the output features from the backbone network’s P3, P4, and P5 through the SSFF module. This aims to effectively merge feature maps at varying spatial scales to capture types of diverse sizes and morphologies. In SSFF, the feature maps of P3, P4, and P5 are scaled down to the same size and then stacked on top of each other by upsampling. These are then fed into 3D convolution^27^ to combine features from different scales.

The TFE module, on the other hand, makes it easier to find small and dense targets by putting together large, medium, and small features in space. Subsequently, the detailed features of the TFE module are integrated into each feature branch through the PANet structure, combined with the multi-scale information of the SSFF module in the P3 branch.

The structure of ASF-Sim-YOLO is shown in Fig. 1. Given that the P3 layer has demonstrated high performance in image division, the introduction of a higher resolution P2 layer can further enhance the recognition of small-sized targets.

Because small targets are often visually mixed with backgrounds, the high-resolution P2 layer can help models better distinguish the subtleties between targets and complex backgrounds. In this paper, adding the P2 detection layer to the detector header’s last layer allows YOLOv8 to more effectively detect smaller-scale escalator step defects. Small targets typically occupy fewer pixels in an image, making them more likely to be missed or misjudged. The dedicated P2 layer in YOLOv8 enhances the sensitivity of small target detection, improving overall accuracy. The introduction of P2 in BiFPN enables bidirectional information propagation across different resolution levels, allowing for better fusion of multi-scale information. This enhancement helps the model understand objects of various sizes more comprehensively and improves detection performance for multi-scale objects. Additionally, it enhances context understanding, reducing both false positives and missed detections. The schematic of the P2 small target detection layer is shown in Fig. 2.

**Fig. 3:**
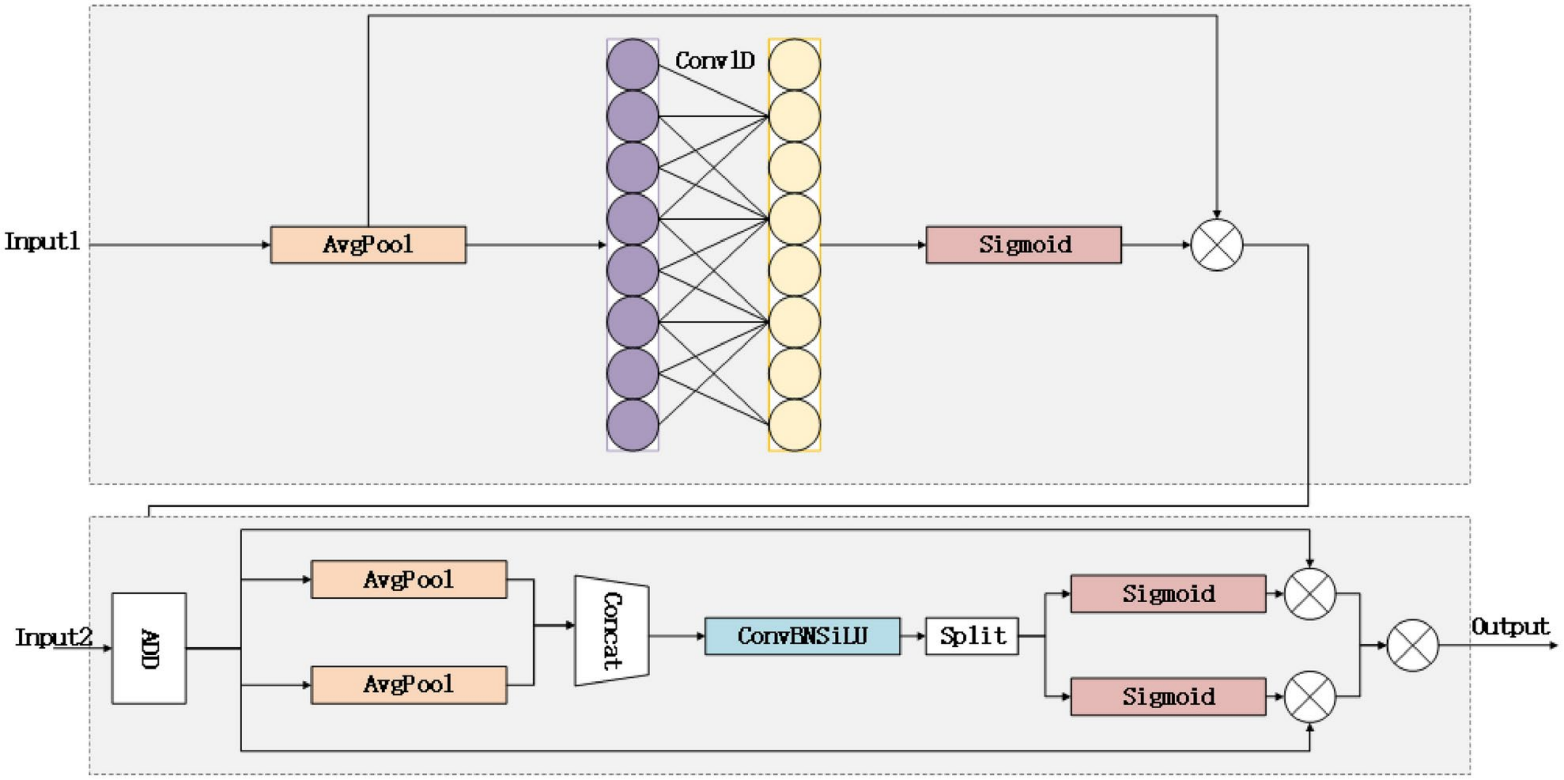
CPAM module structure.

**Fig. 4:**
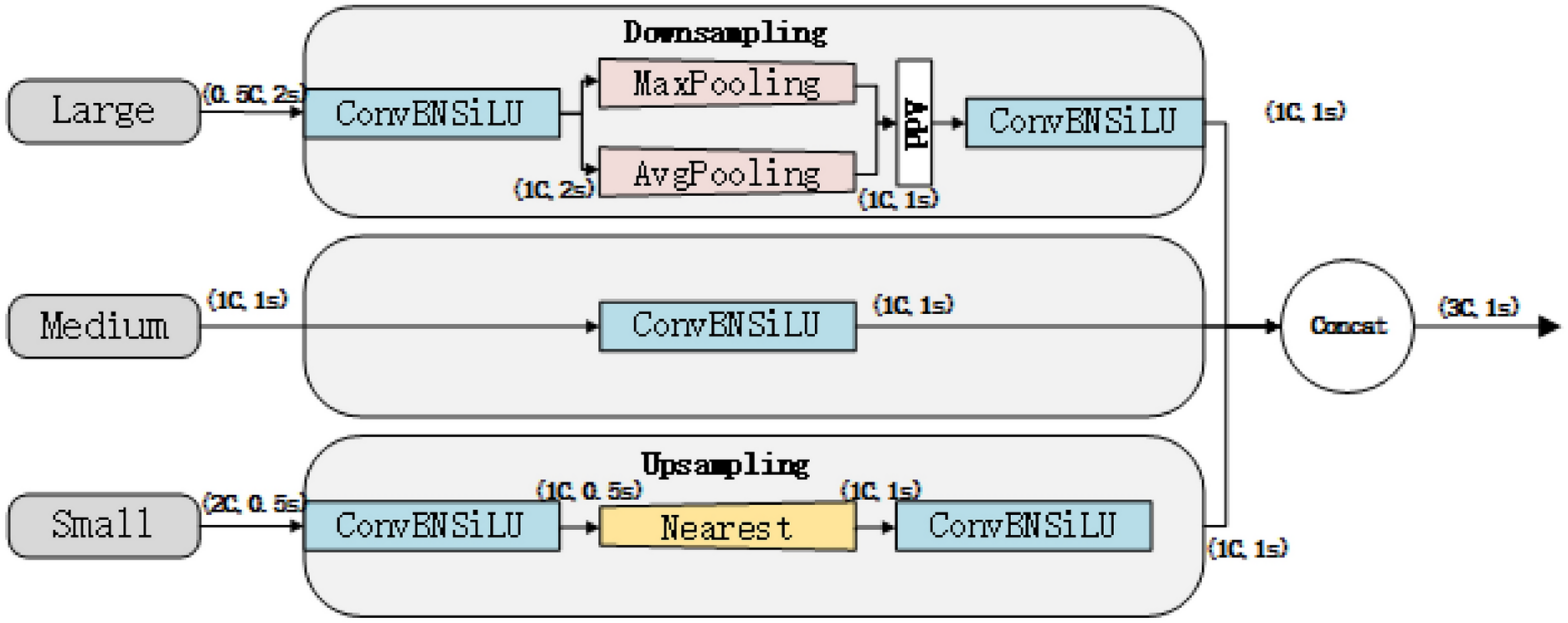
TFE module structure.

Figure 3 illustrates the structure of the CPAM module. Input 1 is the channel attention network, representing the feature map after PANet processing, which includes detailed TFE features. The SENet channel attention block performs independent global average pooling on each channel, followed by two fully connected layers and a nonlinear Sigmoid function to generate channel weights. Figure 4 depicts the TFE module structure. Before feature coding, the number of feature channels is adjusted to align with the main scale feature. Large feature maps undergo convolution, reducing the number of channels to 1, and employing a mixed structure (max pooling + average pooling) for downsampling. This approach helps maintain the validity and diversity of high-resolution features and images. For small feature maps, a convolutional module adjusts the number of channels, followed by nearest neighbour interpolation for upsampling, which preserves the rich local features of low-resolution images while preventing the loss of small target features. Finally, the large, medium, and small feature maps are convolved to the same size and then concatenated by channel.

**Fig. 5:**
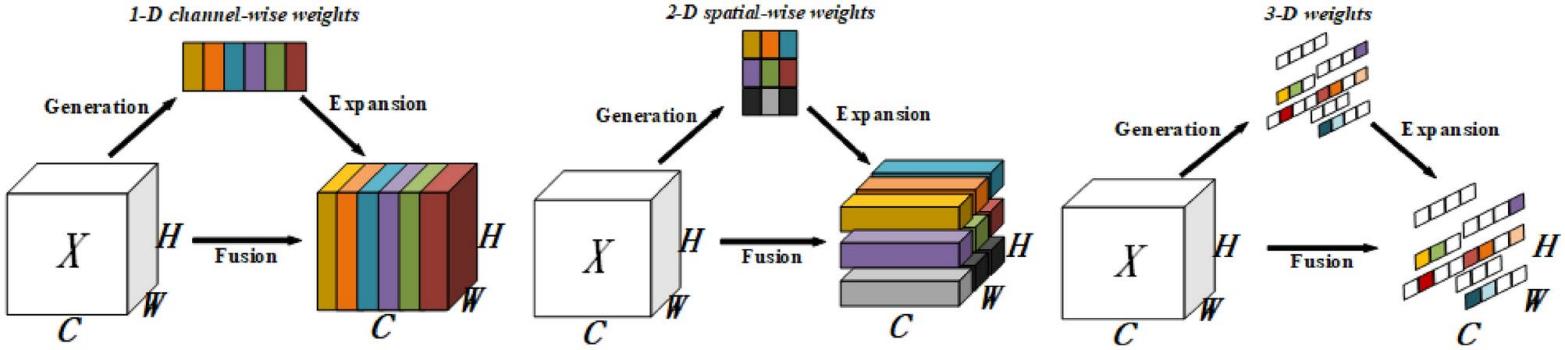
Different weightings of SimAM.

This paper introduced a neuroscience-based SimAM^28^ module to improve the network’s ability to find defects in steps and reduce background noise that can make defect detection harder. By integrating SimAM with SPPF, this combination synergises the strengths of both. Initially, SPPF extends the feature’s receptive domain and captures information at various scales^29^ through pooling operations. Subsequently, SimAM refines the representation of these features by assigning weights to each pixel based on their importance, thereby augmenting the model’s capacity to capture crucial information. This heightened attention to detail contributes to enhanced detection accuracy for escalator step defects. The schematic diagram of SPPF and SimAM fusion is shown in Fig. 5.

**Fig. 6:**
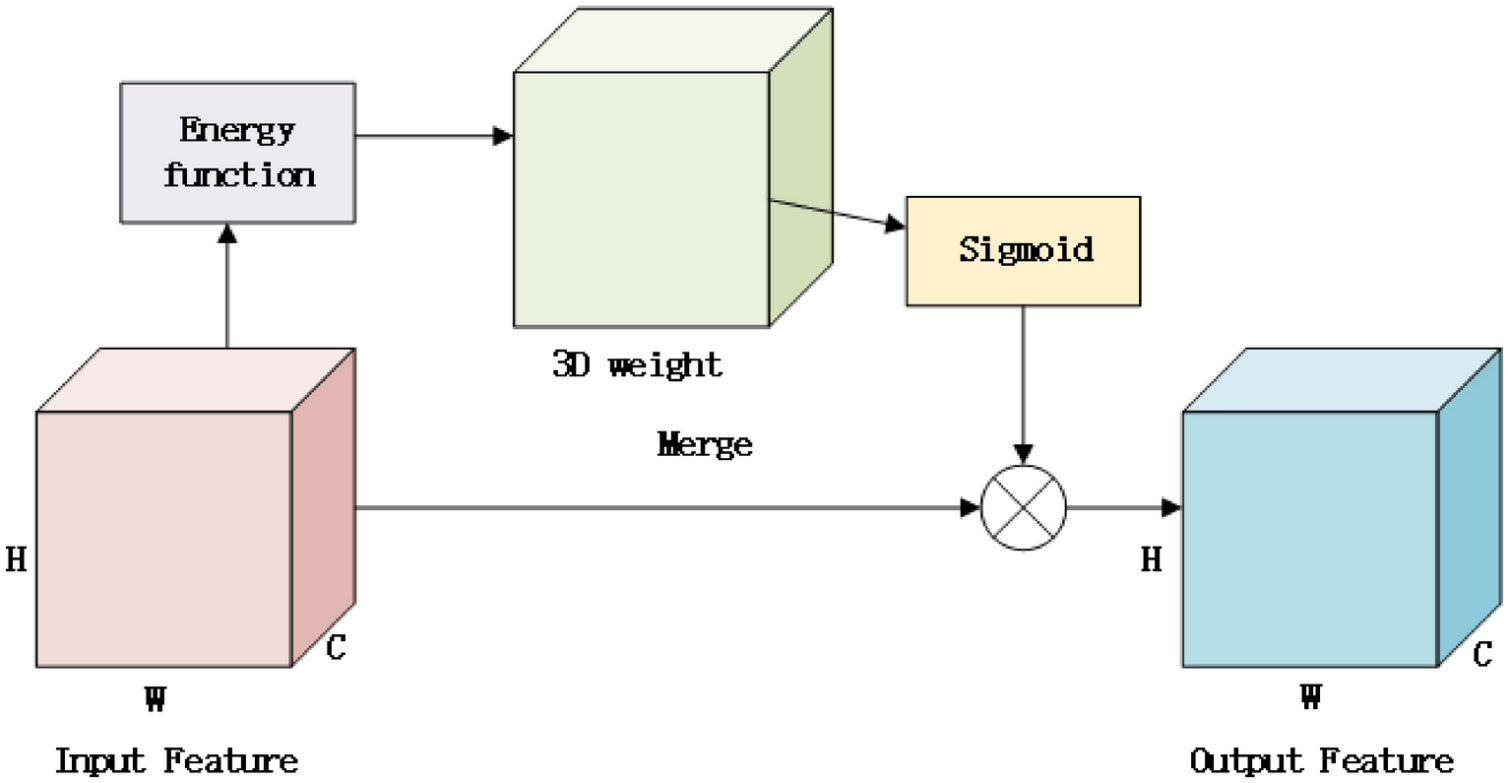
Input and output streams of SimAM.

Figure 6 shows that SimAM receives input features from the previous layer or network, and the input features are processed through a set of three-dimensional weights, which represent a convolution kernel for extracting different aspects of the input features. The over-weighted features undergo some transformations, such as convolution operations, to generate intermediate features. The intermediate features are processed by the Sigmoid activation function, which has an output range between 0 and 1, which is usually used to indicate the importance or activation of each feature, and the output of the Sigmoid function serves as the attention weights, which indicate which parts of the input features are important. The attention weight is merged with the original input features. This merging process may involve weighted summing or other operations to enhance important parts of the input features. The combined results serve as output features that contain information enhanced by attention mechanisms that can be used at the next layer of the network.

The attention mechanism emulates the brain’s selective focus on specific regions. Historically, most commonly used attentions were either single-channel or spatial, with few mechanisms simply parallelising or serialising channels and spaces. However, the brain’s attention isn’t as straightforward; it integrates both channel and spatial attention, working in tandem. Usually, the attentional module boosts outputs from the layer before it. It does this by creating one- or two-dimensional spots along a channel or in space, and treating neurons in these spots the same.

The core idea of SimAM is based on the local self-similarity of images. In an image, there is usually a strong similarity between adjacent pixels, while the similarity between distant pixels is weak. SimAM uses this property to generate attention weights by calculating the similarity between each pixel in the feature map and its neighbours.

Contrary to existing mechanisms, the parameter-free attention mechanism (SimAM) doesn’t focus solely on channels and space. Rather than adding extra parameters to the original model, 3D attention weights are directly inferred within one layer of the network model. The structure of SimAM is depicted in Fig. 5, progressing from left to right with 1D, 2D, and 3D weights. Channel-based attention treats channels differently but locations equally, while spatial-type attention treats locations differently but the channels equally. Single-type attention mechanisms may constrain feature learning, whereas 3D weights demonstrate superior performance compared to 1D and 2D weights.

The Parameter-free The attention mechanism is based on neuroscience theory, enabling neurons to be assigned a unique weight. The principal expression is shown below.

1 $$\begin{aligned} {\tilde{x}} = sigmoid\left(\frac{1}{E}\right)\odot X \end{aligned}$$

where *E* denotes the grouping across channels and spatial dimensions, and the activation function *sigmod* prevents excessive values *E* from affecting the relative importance of each neuron. Characteristics of the input $X\in R^{C\times H\times W}$.

### Replacement of loss function

There are some challenges in the dataset such as small and dense defects at the far end of the image, defects at the edges of the image, etc. These situations can lead to the loss of important step defect features during the feature extraction process, making the detection accuracy of step defects decrease. To address this issue, this paper proposed the use of NWD as an alternative to the default CIoU of YOLOv8 to improve the detection accuracy for small targets of step defects.

NWD (Normalised Wasserstein Distance) is a new way to find escalator defects that is meant to fix problems with existing ones, like how sensitive they are to small changes in object position and how they might not work as well with anchor-based detectors. Some evaluation metrics, like the intersection-over-union ratio (IoU), can be affected by small changes in where objects are located. This can cause detectors that use anchors to work less well. Unlike the IoU, NWD shows the bounding box as a 2D Gaussian distribution and figures out how similar they are. This makes it less sensitive to small objects moving out of place.

Because escalator step defects frequently exhibit small sizes and positional deviations, traditional IoU metrics may inadequately assess detection results. The inclusion of NWD can improve the detection algorithm’s performance and robustness by accurately quantifying the similarity between detected step defects and real defects. NWD, a metric tailored for tiny objects based on the Wasserstein distance, involves two stages in its process:

1. Modelling the bounding box as a 2-dimensional Gaussian distribution For tiny objects, they tend to have some background pixels in their bounding boxes because most real objects are not strictly rectangular. In these bounding boxes, foreground and background pixels are concentrated at the centre and boundary of the bounding box, respectively. To better describe the weights of different pixels in the bounding box, the bounding box can be modelled as a two-dimensional (2D) Gaussian distribution, where the centre pixel of the bounding box has the highest weight, and the pixels’ importance decreases from the centre to the boundary. Specifically, for the horizontal bounding box $R=(C_x, C_y, w, h)$, where $C_x, C_y, w$ and *h* denote the centre coordinates, width and height, respectively. The model is a two-dimensional Gaussian distribution $N(\mu , \sum )$.
  2 $$\begin{aligned} \mu =\begin{bmatrix} C_x\\ C_y\end{bmatrix}, \sum =\begin{bmatrix} \frac{w ^{2}}{4} & 0 \\ 0& \frac{h^{2}}{4} \end{bmatrix} \end{aligned}$$
2. Calculate and normalise the Wasserstein distance definition We define the second-order Wasserstein distance between $\mu _{1}=N(m_{1}+\sum _{t}$ and $\mu _{2}=N(m_{2}+\sum _{2})$ 2D Gaussian distributions as follows:
  3 $$\begin{aligned} W_{2}^{2}(\mu _{1}-\mu _{2})=\Vert m_{1}-m_{2}\Vert _{2}^{2}+\Vert \Sigma _{1}^{\frac{1}{2}}-\Sigma _{2}^{\frac{1}{2}}\Vert _{F}^{2} \end{aligned}$$
  where $\Vert .\Vert _{F}$ denotes the number of paradigms, $W_{2}^{2}$ denotes the distance measure, $m_{1}$, $m_{2}$ denote the mean vector, and $\sum _{1},\sum _{2}$ denote the covariance matrix. Thus, for 2 bounding boxes A and B, where the bounding box $A=(Cx_{a},Cy_{a}, w_{a}, h_{a})$, $B=(Cx_{b}, Cy_{b}, w_{b}, h_{b})$, The second-order Wasserstein distances of the two-dimensional Gaussian distributions $N_a$ and $N_b$ of the bounding box are:
  4 $$\begin{aligned} W_2^2(N_a,N_b)=\left\| \left(\left[ cx_a,cy_a,\frac{w_a}{2},\frac{h_a}{2}\right] ^T,\left[ cx_b,cy_b,\frac{w_b}{2},\frac{h_b}{2}\right] ^T\right)\right\| _2^2 \end{aligned}$$
  *NWD* is calculated as follows:
  5 $$\begin{aligned} NWD(N_a,N_b)=\exp \left( -\frac{\sqrt{W_2^2(N_a,N_b)}}{c}\right) \end{aligned}$$
  Where *Na* and *Nb* are Gaussian distribution models for the predicted and real boxes respectively, $W_{2}^2$ is the calculation of the second-order Wasserstein distance between the two Gaussian distributions, and *C* is a constant used to normalize the distance values.

### Model channel pruning

**Fig. 7:**
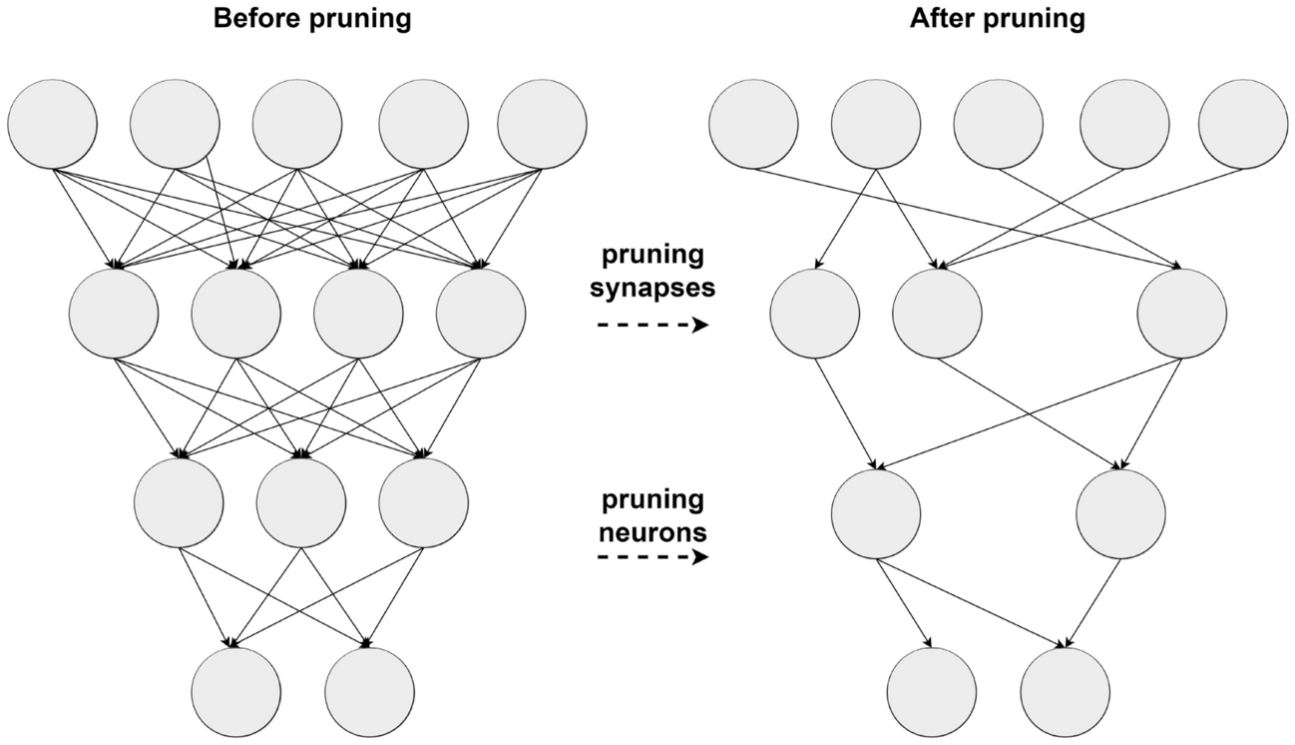
LAMP pruning process schematic.

To meet the challenge of maintaining accuracy while reducing model size for lightweight deployment, both module improvement and model compression are essential. Module enhancement alone may not suffice, necessitating techniques like pruning where unimportant channels are removed. As shown in Fig. 7. This method mainly targets the convolutional layer in the convolutional neural network (CNN), because the convolutional layer usually contains a large number of parameters and is the most resource-intensive part of the model.

The LAMP (Layer-Adaptive Magnitude-based Pruning)^30^ score introduces a novel computational method for weight calculation and pruning. It operates by computing the square of the weight magnitude for the target connection and normalising it against the sum of the squared weight magnitudes of all “surviving weights” within the same layer. The specific formula for the score is as follows:

6 $$\begin{aligned} S(u;W_t)=\frac{(W_t[u])^2}{\sum _{v*u}(W_t[v])^2} \end{aligned}$$

wherein $W_t$ denotes a weight, $W_t[u]$ and $W_t[v]$ denote an item *W* mapped by index *u* and *v*, *u* and *v* denote an index mapping corresponding to the weights according to sorting in ascending order, respectively.

This scoring mechanism enables efficient pruning by identifying and removing less crucial connections while preserving important ones, thus reducing model complexity without significantly sacrificing accuracy.

The advantage of LAMP pruning method is that at least one weight is reserved in each layer, which will not cause the phenomenon of no weight in some layers. Moreover, this weight is a relatively important weight for the extraction of escalator step defect features. The pruning method is shown in formula 7.

7 $$\begin{aligned} {\begin{bmatrix}u_1\\ u_2\\ u_3\\ \cdots \end{bmatrix}\begin{bmatrix}\nu _1\\ \nu _2\\ \nu _3\\ \cdots \end{bmatrix}}\rightarrow \begin{bmatrix}\frac{u_1^2}{u_1^2}\\ \frac{u_2^2}{u_1^2+u_2^2}\\ \frac{u_3^2}{u_1^2+u_2^2+u_3^2}\end{bmatrix}\begin{bmatrix}\frac{v_1^2}{v_1^2}\\ \frac{v_2^2}{v_1^2+v_2^2}\\ \frac{v_3^2}{v_1^2+v_2^2+v_3^2}\end{bmatrix}\rightarrow \begin{bmatrix}\frac{u_1^2}{u_1^2}& \frac{v_1^2}{v_1^2}\\ \frac{u_2^2}{u_1^2+u_2^2}& \phi \\ \phi & \phi \\ \cdots & \cdots \end{bmatrix} \end{aligned}$$

Formula 7 is the weight size, LAMP fraction and dynamic pruning from left to right.

## Experimental studies

In order to validate the effectiveness and reasonableness of ASF-Sim-YOLO for escalator step defect detection, two sets of experiments were conducted using the enhanced dataset.

1. Ablation experiment The proposed ASF-Sim-YOLO has undergone several stages of improvement. In order to verify the impact of each improvement strategy on the model detection performance, this study designed ablation experiments on the expanded dataset.
2. Comparative experiment In order to further validate the detection performance of ASF-Sim-YOLO on the dataset, we selected five currently best performing lightweight object detection models for comparison.

### Dataset

**Fig. 8:**
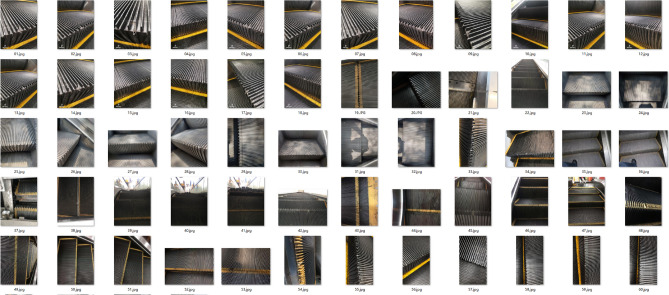
Escalator step defects dataset.

**Fig. 9:**
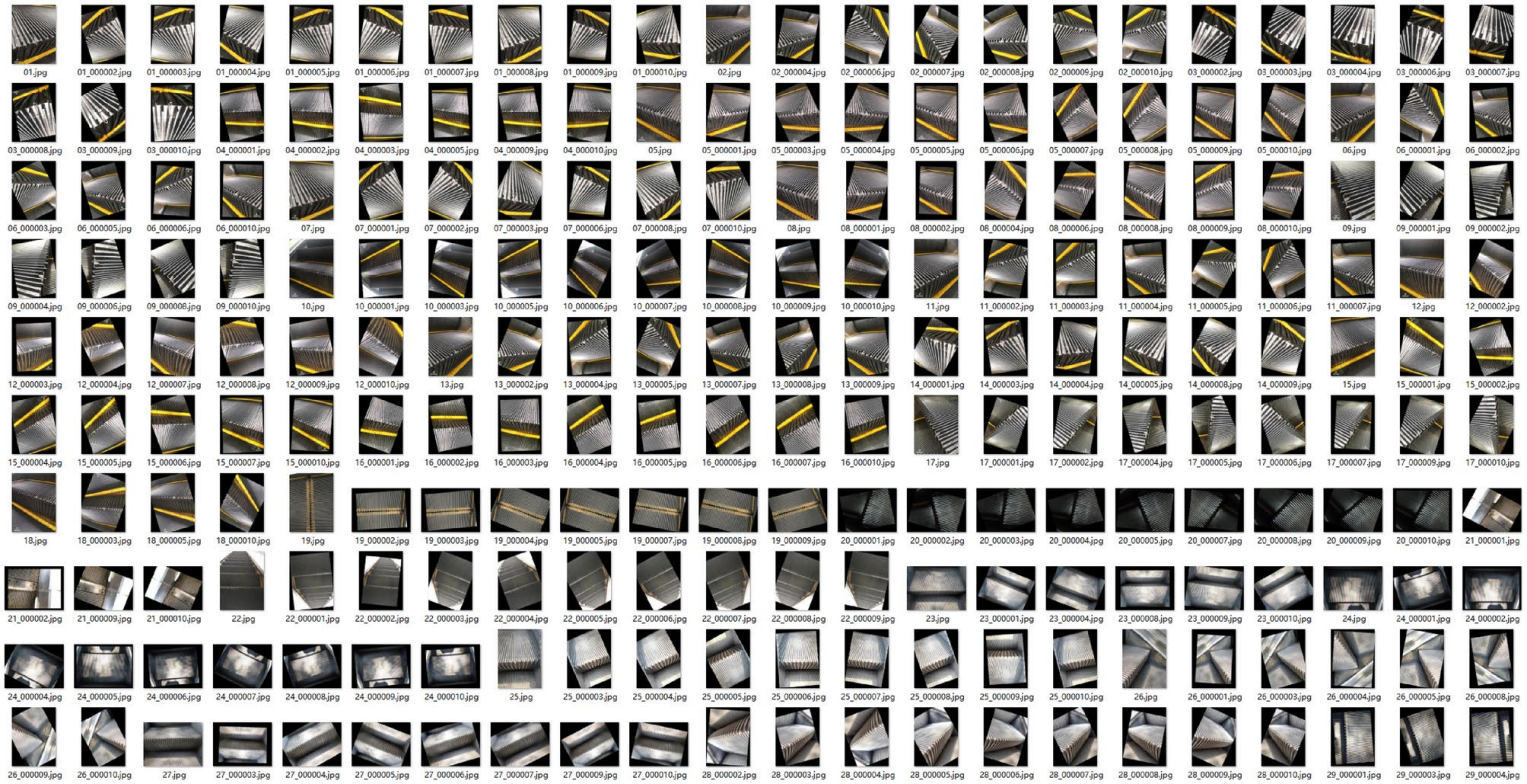
Data enhancement.

The dataset, shown in Fig. 8, consists of an initial dataset of 67 images, all of which depict escalator missing teeth defects. Given the limited size of the original dataset, we adapted it to expand it. Image enhancement techniques included random rotation, horizontal panning, brightness adjustment, and noise addition. With these modifications, the expanded dataset contained 737 images.

The enhanced results are illustrated in Fig. 9 By adding more samples to the dataset and making it more diverse, the limitations caused by the small number of samples can be lessened. This also helps keep the dataset from becoming too fit, which improves the network model’s ability to generalise.

To ensure data randomness, the dataset is randomly divided into training, validation, and test sets, with a proportion of 70% for the training set, 20% for the validation set, and 10% for the test set. This division scheme helps ensure that the model is trained on a diverse range of data while also providing separate subsets for evaluating performance during training and the final assessment.

### Experimental setup

#### Experimental platform

To enhance the efficiency of the ASF-Sim-YOLO algorithm, all training and testing tasks described in this paper were executed on a single GPU system. The system specifications include: CPU: 13th Gen Intel (R) CoreTM i5-13600KF @ 3.50 GHz. RAM: 32 GB. GPU: NVIDIA GeForce RTX3070Ti-8G. Python interpreter Version: 3.8.16. Pytorch version: 1.13.1. Accelerated environment: CUDA 11.7. OS: Windows 11 Professional 22H2.

Because of the paper’s lightweight design, only the smaller-scale models of the YOLO series are compared. The algorithms used for comparison are YOLOv3-tiny, YOLOv5s, YOLOv6, YOLOv8n, and YOLOv8s.

In the experiments, identical hyper-parameters are applied across all the deep learning methods utilised. While the fine-tuning of hyper-parameters remains an ongoing challenge requiring substantial research effort, the primary focus of this paper lies in the development of a new defect detection model featuring a specially designed architecture rather than in hyper-parameter optimisation. The hyper-parameter settings of the model in this paper are detailed in the accompanying Table 1.

The number of model layers, number of parameters, computational complexity (GFLOPs), mAP@0.5, and mAP@0.5-0.95, along with other metrics, were estimated during the model training process to showcase the performance of the model.

**Table 1:** Hyper-parameter setting.

Hyperparameter	Value
Momentum	0.937
Epochs	100
BatchSize	16
CloseMosaic	10
Workers	8
Optimizer	SGD
WeightDecay	0.0005

Momentum is a technique that helps accelerate convergence during gradient descent by introducing an exponentially weighted moving average of the previous gradient into the parameter update. This can help avoid local optima to some extent and reduce the phenomenon of gradient oscillation. 0.937 is an empirical value that has been verified through extensive experiments. The value of momentum is usually between 0.9 and 0.99, and a value that is too low may not fully utilise the gradient information from the previous iteration, while a value that is too high may cause the model to skip the optimal solution. 0.937 was found to achieve a good balance between convergence speed and stability in YOLO. The training epochs is based on the number of pictures in the data set. After 100 training rounds, the decline rate of the training loss function becomes very small, and after 100 training rounds, the loss decline is very small. Batch Size According to the GPU video memory capacity setting, the equipment used for training is 3070Ti 8GB. Through experiments, it is found that the Batch Size set to 16 is the best number to balance time and performance Since the model in this paper performs well on the data set, it is beneficial to turn off Mosaic enhancement. Workers Index Indicates the number of CPU threads during loading, which affects the memory usage. The value set to 8 is the result of a large number of experiments. SGD is widely used in training deep learning models, especially convolutional neural networks (such as YOLO). It is known for its stability and generality. It can handle large-scale datasets especially in environments with limited computing resources. In such cases, the training efficiency and stability of SGD are more prominent. Compared to adaptive optimisers such as Adam, SGD is more helpful for model generalisation. Although adaptive optimisers converge faster during the initial training phase, they may result in poorer performance of the model on the test set. YOLO uses SGD to take advantage of the batch gradient information and gradually optimise the model parameters when processing batches of images, so it chooses SGD. Weight-decay is used neither to increase convergence accuracy nor to increase convergence speed; its ultimate purpose is to prevent over-fitting. A setting of 0.0005 is the result of extensive experiments.

#### Performance metrics

In the YOLO series of models, the metrics for evaluating network performance mainly include the following: precision (P), recall (R), and mean average precision (mAP). The experiments typically utilise two metrics, mAP@0.5 and mAP@0.5:0.95, as performance references. Among them, mAP@0.5 denotes the mean average precision at the IoU threshold of 0.5, while mAP@0.5:0.95 denotes the mean average precision in the range of IoU gradually increasing from 0.5 to 0.95. The formulas for these metrics are given below:

8 $$\begin{aligned} P= & \frac{T_P}{T_P+F_P}\times 100\% \end{aligned}$$

9 $$\begin{aligned} R= & \frac{T_P}{T_P+F_N}\times 100\% \end{aligned}$$

10 $$\begin{aligned} AP= & \int _{0}^{1}PRdr \times 100\% \end{aligned}$$

11 $$\begin{aligned} mAP= & \frac{\sum _{i=1}^{n}AP_{i}}{n}\times 100\% \end{aligned}$$

In deep learning, $T_P$ represents the number of correctly predicted positive samples, $F_N$ represents the number of incorrectly predicted negative samples, and $F_P$ represents the number of incorrectly predicted positive samples. *n* represents the number of data classes in the dataset, which in this paper was taken as 1. There is one class of step surface defect, i.e., missing teeth. When determining how to balance the model’s lightweight and performance, the number of parameters and computational complexity must also be considered *FPS* is used to measure the processing speed of the model, and the larger the FPS value of the model indicates the faster the detection speed. Algorithmic model’s memory occupation is reflected in the number of parameters it uses.

12 $$\begin{aligned} FPS= & \frac{1}{T} \end{aligned}$$

13 $$\begin{aligned} Parameters= & (K_h\times K_w\times C_{in})\times C_{out}+C_{out} \end{aligned}$$

where $C_{in}$ and $C_{out}$ denote the number of channels of the input and output feature maps, $K_w$ and $K_h$ denote the width and height of the convolution kernel, respectively.

## Results

### Ablation experiment

In this study, we used the YOLOv8n model to do a number of improvement and ablation experiments to see how different combinations affected its ability to find targets. GFLOPS measures the number of forward propagation operations in the neural network, with lower GFLOPS indicating faster computation speed. The number of parameters quantifies the parameters included in the neural network, with fewer parameters indicating a smaller model size and easier deployment. The ablation experiments are summarised in Table 2.

Before proceeding with the improvements, we initially evaluated the original YOLOv8n model as a benchmark.

The first set of improvements is based on the ASF-P2 framework of the original model. The ASF-YOLO module is introduced to enhance detection accuracy for small targets. While the number of parameters is reduced by 17.2% relative to the benchmark model, the computational complexity (GFLOPS) reaches 12.0.

The second set introduces the SimAM attention module, which is a parameter-free attention mechanism. This improvement improved mAP@0.5 by 4.5% and mAP@0.5:95% by 0.7% without increasing the number of parameters or computational complexity.

The third set introduces the NWD loss function, which improves the design of the loss function without affecting the number of parameters or computational complexity of the model. The results show an improvement of 8.1% for mAP@0.5 and 7.8% for mAP@0.5:95

The fourth set simultaneously improved the ASF-P2 framework and introduced the SimAM attention module. This combination improved mAP@0.5 by 12.6% and mAP@0.5:95% by 16%, reduced the number of parameters by 17.2%, and increased the computational complexity to 12.0 GFLOPS.

The fifth set introduces the SimAM attention module and the NWD loss function. Without increasing the number of parameters and computational complexity, mAP@0.5 improves by 6.3%, and mAP@0.5:95% improves by 8.7%.

The sixth set introduced both the SimAM attention module and the NWD loss function in the ASF-P2 framework. The results show that mAP@0.5 is improved by 11.9%, mAP@0.5:95% is improved by 15.3%, the number of parameters is reduced by 17.2%, and the computational complexity is improved to 12.0 GFLOPS.

Finally, the seventh set combined the ASF-P2 framework with the SimAM attention module and the NWD loss function. Relative to the benchmark model, mAP@0.5 improved by 13.9%, and mAP@0.5:95% improved by 16.2%.

**Table 2:** Ablation experiment.

No.	ASF-P2	SimAM	NWD	mAP@0.5	mAP@0.5:95%	Parameters	GFLOPS
0	$\times$	$\times$	$\times$	0.793	0.425	3005843	8.1
1	$\checkmark$	$\times$	$\times$	0.880	0.476	2489300	12.0
2	$\times$	$\checkmark$	$\times$	0.829	0.428	3005843	8.1
3	$\times$	$\times$	$\checkmark$	0.857	0.458	3005843	8.1
4	$\checkmark$	$\checkmark$	$\times$	0.893	0.493	2489300	12.0
5	$\times$	$\checkmark$	$\checkmark$	0.843	0.462	3005843	8.1
6	$\checkmark$	$\times$	$\checkmark$	0.887	0.490	2489300	12.0
7	$\checkmark$	$\checkmark$	$\checkmark$	0.903	0.494	2489300	12.0

### Comparative experiment

**Fig. 10:**
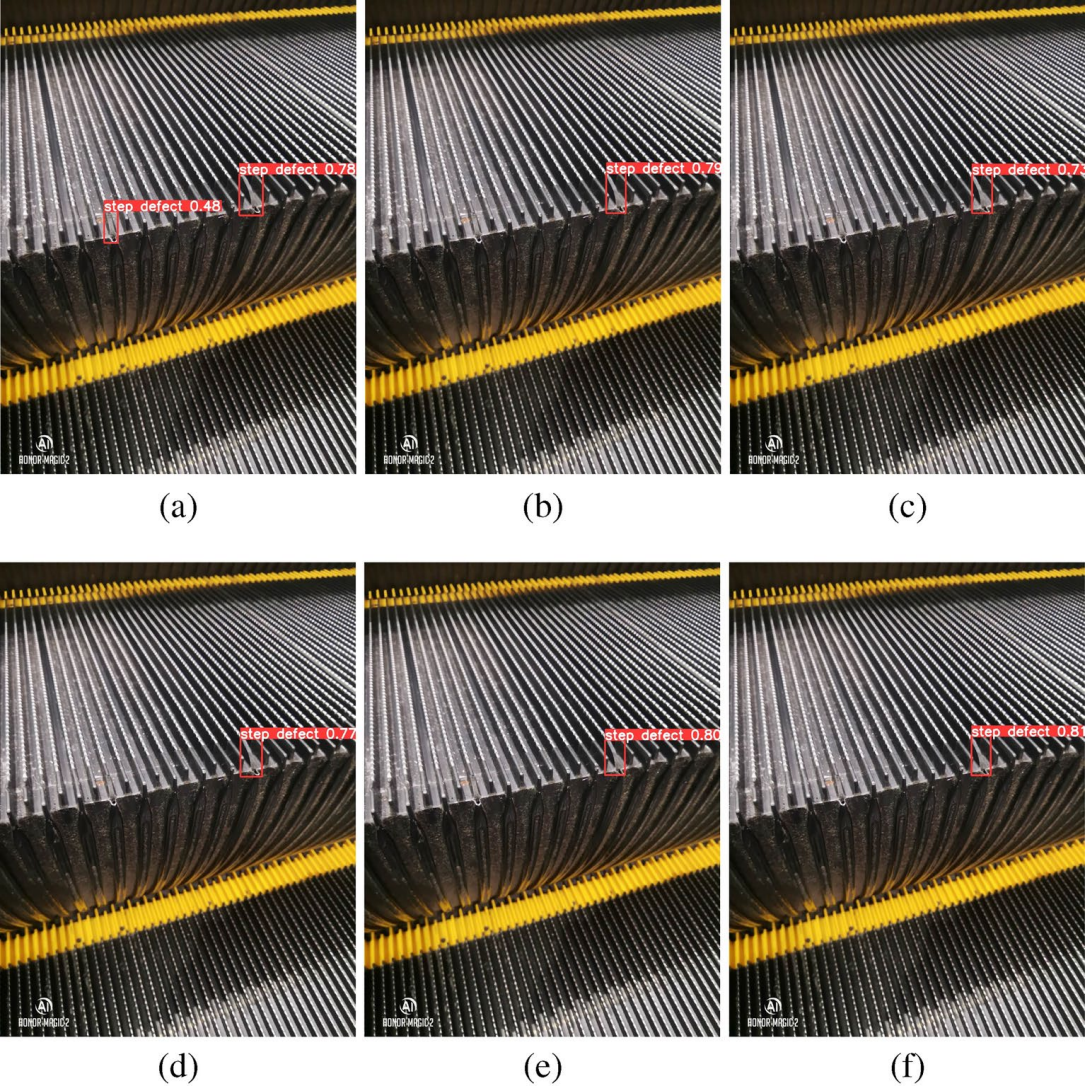
(**a**), (**b**), (**c**) , (**d**) , (**e**) , (**f**) are ASF-Sim-YOLO, YOLOv3-tiny, YOLOv5s, YOLOv6s, YOLOv8n, YOLOv8s, respectively.

Figure 10a, b, c, d, e, f show the detection results of ASF-Sim-YOLO, YOLOv3-tiny, YOLOv5s, YOLOv6s, YOLOv8n, and YOLOv8s, respectively, and it can be seen that except for ASF-Sim-YOLO, no defects are detected for the rest of the models.

**Fig. 11:**
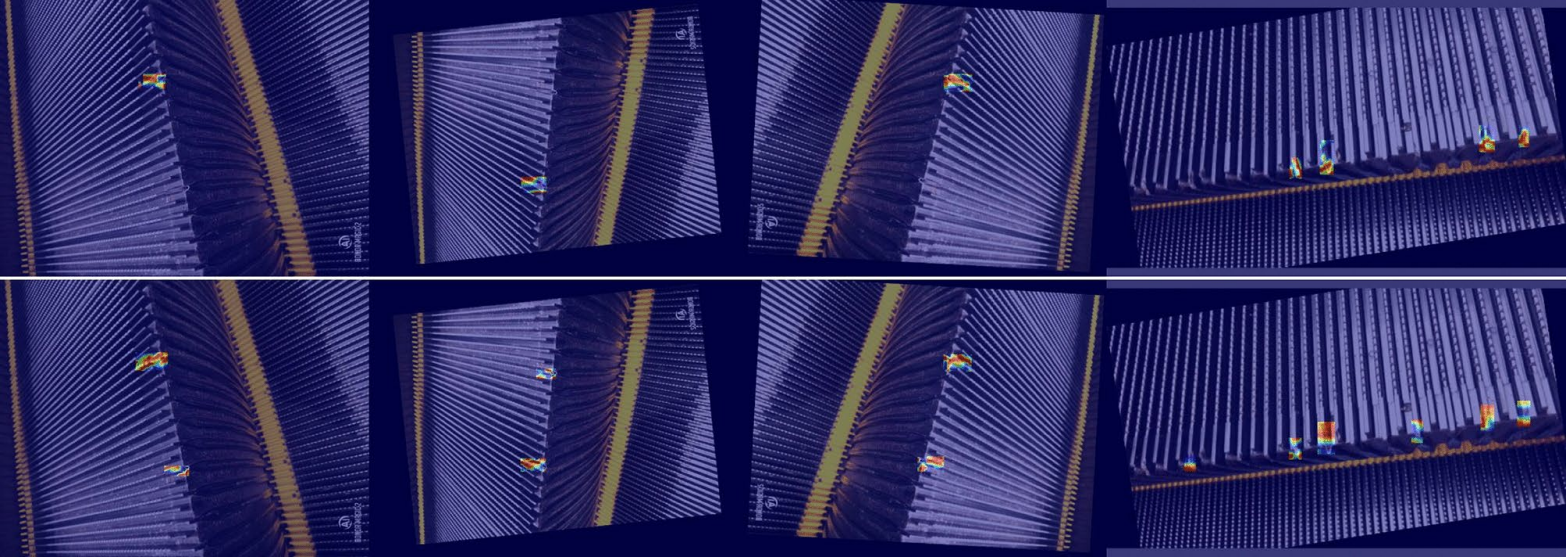
Heat-map of YOLOv8n compared with ASF-Sim-YOLO.

As shown in Fig. 11, the comparison of YOLOv8n with the model proposed in this paper shows that in the four pictures shown in the heat-map, YOLOv8n pays slightly less attention to features than the model proposed in this paper, and some features are missing.

**Fig. 12:**
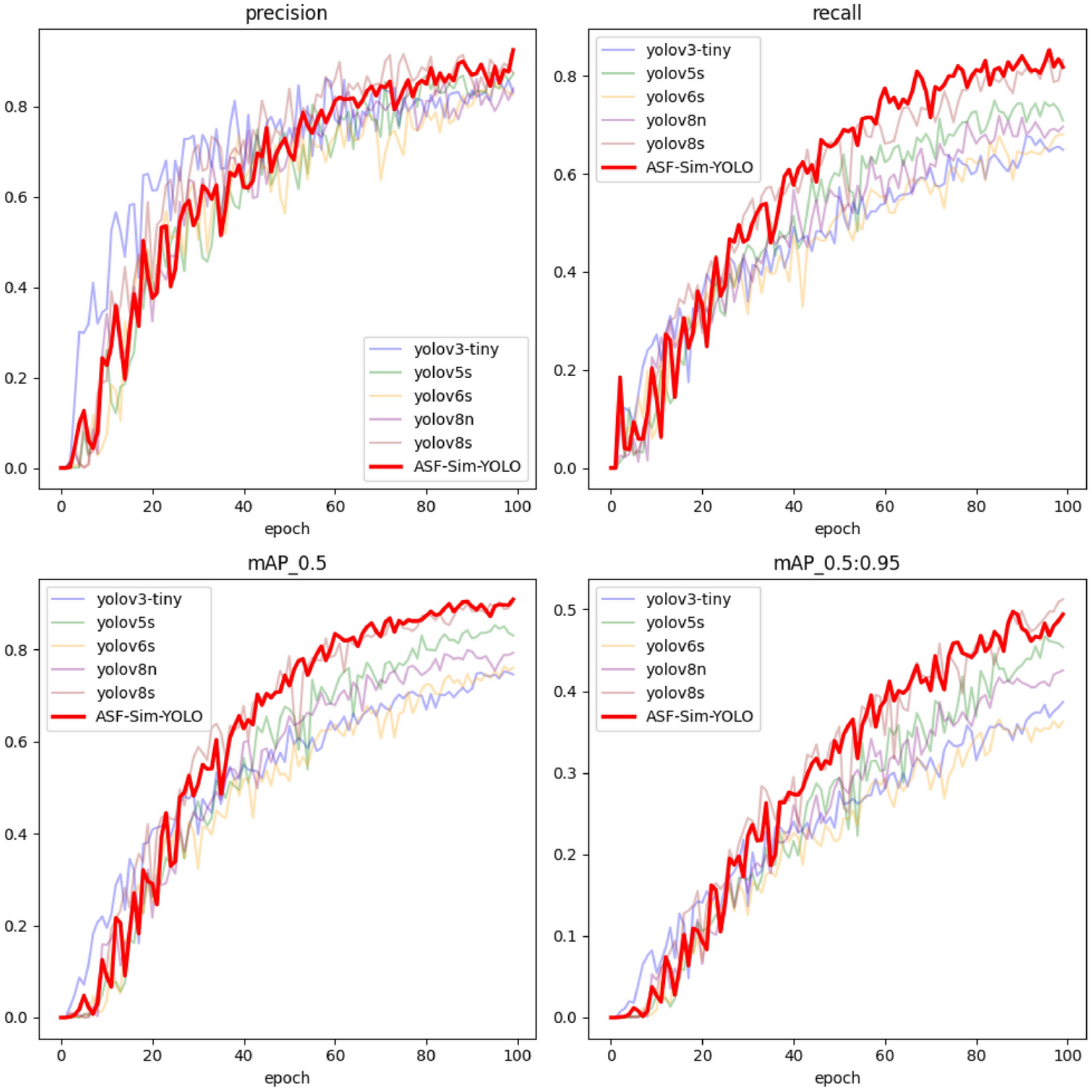
Comparison of training processes.

The training process, illustrated in Fig. 12, demonstrates that the ASF-Sim-YOLO model maintains high accuracy and recall throughout the initial 100 epochs, particularly excelling in precision. This signifies the ASF-Sim-YOLO model’s robust generalisation ability and stability in target detection tasks. Moreover, ASF-Sim-YOLO exhibits superior performance compared to all other models, reaffirming its efficacy in target detection.

From a lightweight design perspective, ASF-Sim-YOLO was compared with commonly used target detection algorithms, namely YOLOv3-tiny, YOLOv5s, YOLOv6s, YOLOv8n, YOLOv8s, RTMDET, TOOD and Faster-RCNN. Under consistent experimental conditions, the comparative results are presented in Table 3. This comparison underscores ASF-Sim-YOLO’s efficiency and accuracy as a target detection method.

**Table 3:** Comparative experiment.

Model	Parameters	GFLOPS	mAP@0.5	mAP@0.5:95%	Epochs
YOLOv3-tiny	12128178	18.9	0.746	0.386	100
YOLOv5s	9111923	23.8	0.853	0.467	100
YOLOv6s	4233843	11.8	0.744	0.365	100
YOLOv8n	3005843	8.1	0.793	0.425	100
YOLOv8s	11125971	28.4	0.906	0.513	100
ASF-Sim-YOLO	2489300	12.0	0.903	0.494	100
RTMDET	4873000	8.025	0.757	0.348	100
TOOD	3201800	172.032	0.777	0.365	100
Faster-RCNN	41348000	182.272	0.870	0.468	100

### Channel pruning

In order to further compress the model, LAMP pruning is performed on the final improved network model. First, the training weight of the improved model was obtained, and LAMP pruning was carried out based on it. The main network feature extraction layer is retained for feature extraction of step defects. Other structures undergo normal pruning. In addition, different Speed-up values will lead to different effects in terms of accuracy, model size and number of model parameters, etc. Therefore, this experiment obtains the optimal pruning model by adjusting the Speed-up values. The results of pruning experiment are shown in Table 4. As can be seen from Table 4, when speed-up is set to 3.0 and 3.5, the size of the model after pruning is 1.04 and 0.87MB, and the detection accuracy is 95.1% and 95.3%, respectively. Compared with the detection accuracy before pruning, the detection accuracy is somewhat increased, and the number of calculated parameters is somewhat decreased. When speed-up is set to 4.0, the size of the model after pruning is 0.8MB and the number of parameters is 205325, and the calculation amount, parameter number and size of the model all decrease significantly. When speed-up is set to a higher value, the accuracy of the model decreases, and the decrease of calculation amount and parameter number becomes not obvious. Therefore, the pruning model with speed-up of 4.0 is selected as the final optimisation model.

**Fig. 13:**
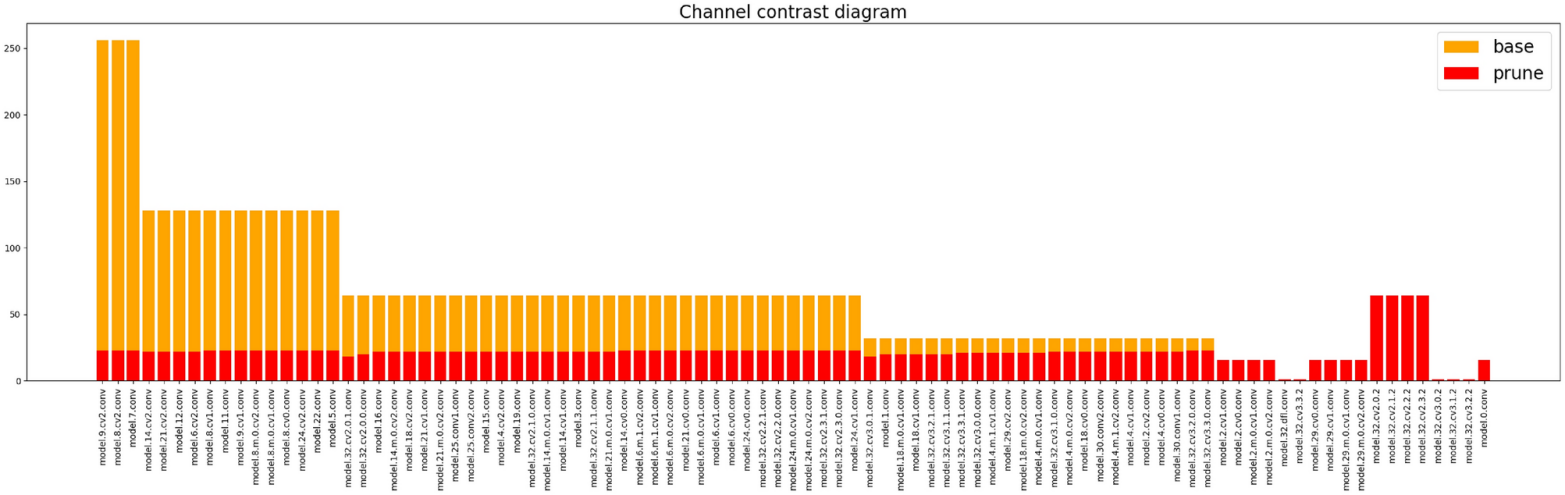
Channel pruning.

The effect of LAMP pruning is shown in Fig. 13. The orange part in the figure represents the base state, and the red part represents the state after pruning.

**Table 4:** Fine-tune experiment.

Speed-up	Parameters	GFLOPS	mAP@0.5	Size
3.0	333672	3.9	0.951	1.04
3.5	273468	3.3	0.953	0.87
4.0	205325	2.8	0.968	0.8
4.5	205325	2.6	0.948	0.741
5.0	178567	2.4	0.944	0.69

As can be seen from Table 5, after model pruning of ASF-Sim-YOLO, the size of the model is reduced to 0.8MB, the real-time detection FPS reaches 575.1, mAP@0.5 increases by 22.1%, and GFLOPS also decreases by 63%, effectively completing the lightweight.

**Table 5:** Comparison of model performance before and after pruning.

Model	Size(MB)	FPS(Batch=16)	mAP@0.5 Enhancement(%)	GFLOPS Drop(%)
ASF-Sim-YOLO	0.8	575.1	22.1	-63

As shown in Table 6, YOLOv3-tiny and YOLOv8s exhibit large model sizes of 23.3M and 21.5M, respectively, whereas YOLOv8n and ASF-Sim-YOLO feature significantly smaller model sizes of 6.2M and 0.8M, respectively. This highlights ASF-Sim-YOLO’s effective model compression technique, resulting in a substantial reduction in model size. In terms of FPS, YOLOv8n and ASF-Sim-YOLO stand out, with rates of 596.7 and 575.1, respectively, indicating their high real-time performance. Despite its smaller size, the YOLOv3-tiny has a slightly lower FPS than other models. Although YOLOv5s has a slightly lower FPS compared to YOLOv8n and ASF-Sim-YOLO, it still achieves a respectable rate of around 315.0 frames per second.

The latency, which represents the time required for model inference, is relatively low for YOLOv8n and ASF-Sim-YOLO, at 0.00168s and 0.00174s, respectively. YOLOv5s exhibits the highest latency of 0.00317s, possibly due to its larger model size.

mAP@0.5 represents the model’s average accuracy for target detection tasks with an IoU threshold of 0.5. ASF-Sim-YOLO outperforms other models with an mAP@0.5 of 0.968, while others achieve scores ranging from 0.746 to 0.906.

GFLOPs represent the number of floating-point operations performed per second. YOLOv8s has the highest GFLOPs of 28.4, while ASF-Sim-YOLO significantly reduces it to 3.0. This indicates ASF-Sim-YOLO’s efficient model design, achieving excellent performance while maintaining low computational complexity.

In summary, the ASF-Sim-YOLO model excels in terms of model size, FPS, latency, mAP@0.5, and GFLOPs, demonstrating high real-time performance and accuracy. It is well-suited for scenarios requiring real-time target detection.

**Table 6:** Model lightweight performance comparison.

Models	Size	FPS	Latency	mAP@0.5%	GFLOPS
YOLOv3-tiny	23.3M	508.2	0.00197s +- 0.00088s	0.746	18.9
YOLOv5s	17.7M	315.0	0.00317s +- 0.00095s	0.853	23.8
YOLOv8n	6.2M	596.7	0.00168s +- 0.00081s	0.793	8.1
YOLOv8s	21.5M	285.1	0.00351s +- 0.00126s	0.906	28.4
ASF-Sim-YOLO	0.8M	575.1	0.00174s +- 0.00084s	0.968	3.0

## Conclusion

In this paper, the original YOLOv8n model was lightened and improved for the escalator step defect detection task to adapt to the limited hardware resources of the detection equipment. An improved YOLOv8n-based defect detection method is proposed. We designed a novel network, ASF-Sim-YOLO, to enhance detection performance. By incorporating the SimAM attention mechanism, the model’s ability to utilise feature information was improved without increasing the parameters, thereby increasing detection accuracy.

Additionally, this study replaced the original CIoU loss function with the NWD loss function, leading to further improvement in detecting defects with more specific locations.

Moreover, channel pruning of the model by weighted model pruning significantly reduced the number of parameters in the model. ASF-Sim-YOLO achieves a balance of performance and lightness, meeting the demand for real-time detection when deployed on mobile devices.

However, while the primary focus of this paper is on detecting missing teeth defects in escalator steps, other types of escalator defects also exist. Obtaining datasets for these other defect types remains challenging. The next goal of this research is to obtain a data set of defects in the comb plate, which is more likely to lead to hazards, such as pulling passenger clothing, shoes, etc. into the gap between the step and the comb plate, causing crushing injuries. Therefore, it is necessary to continue to collect the defect data set of the comb plate, and it is meaningful to input the model in this paper for further training to identify the defects in order to improve the safety of the escalator.
